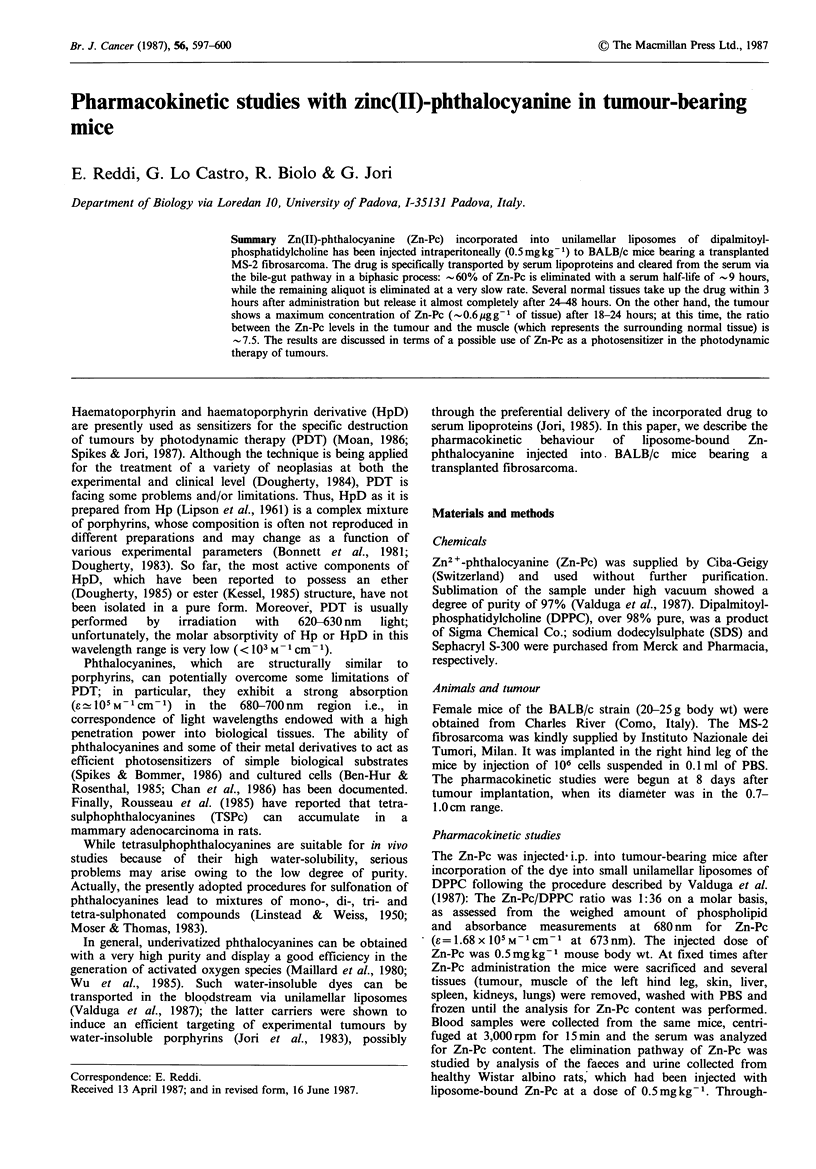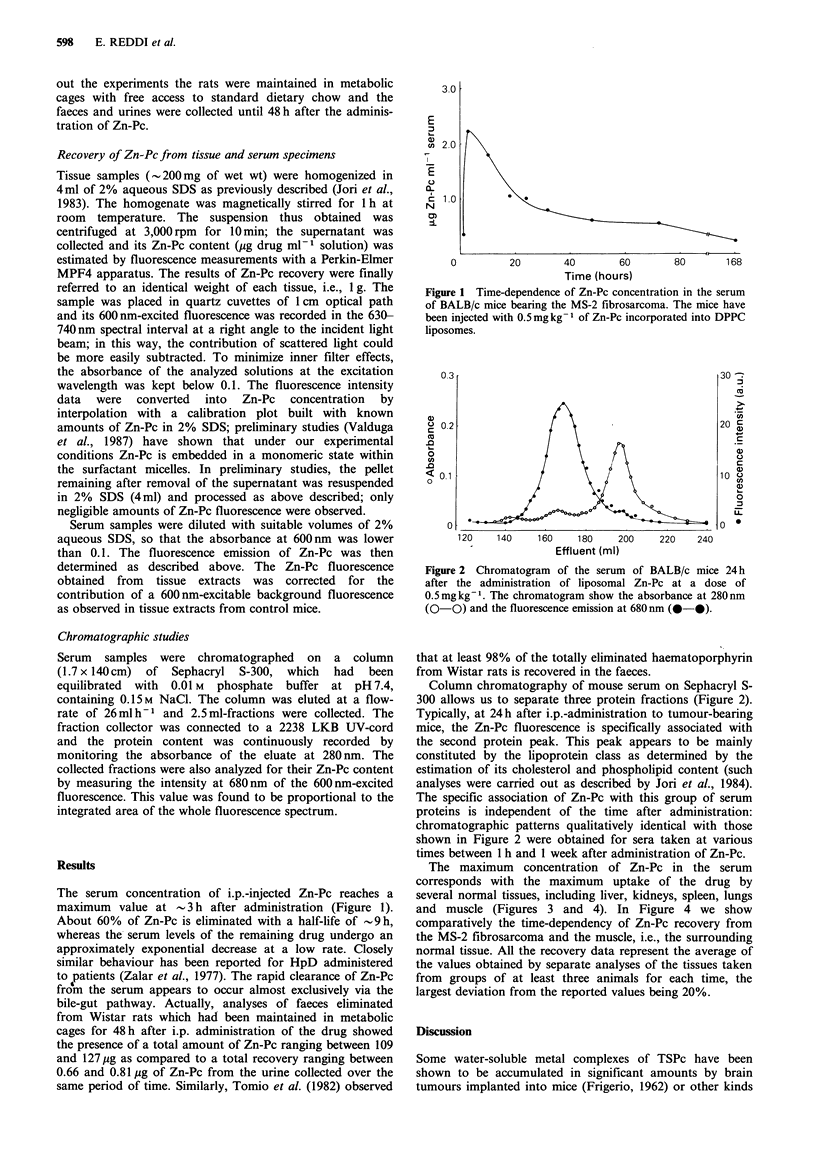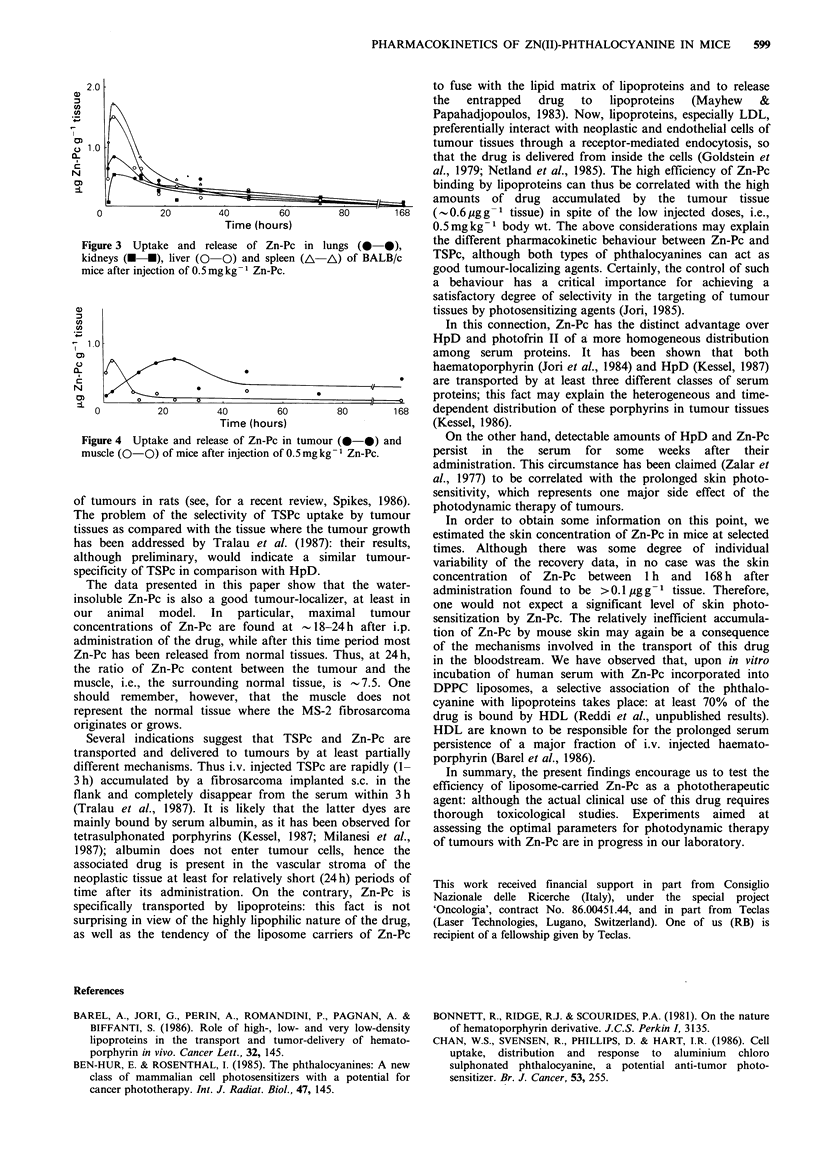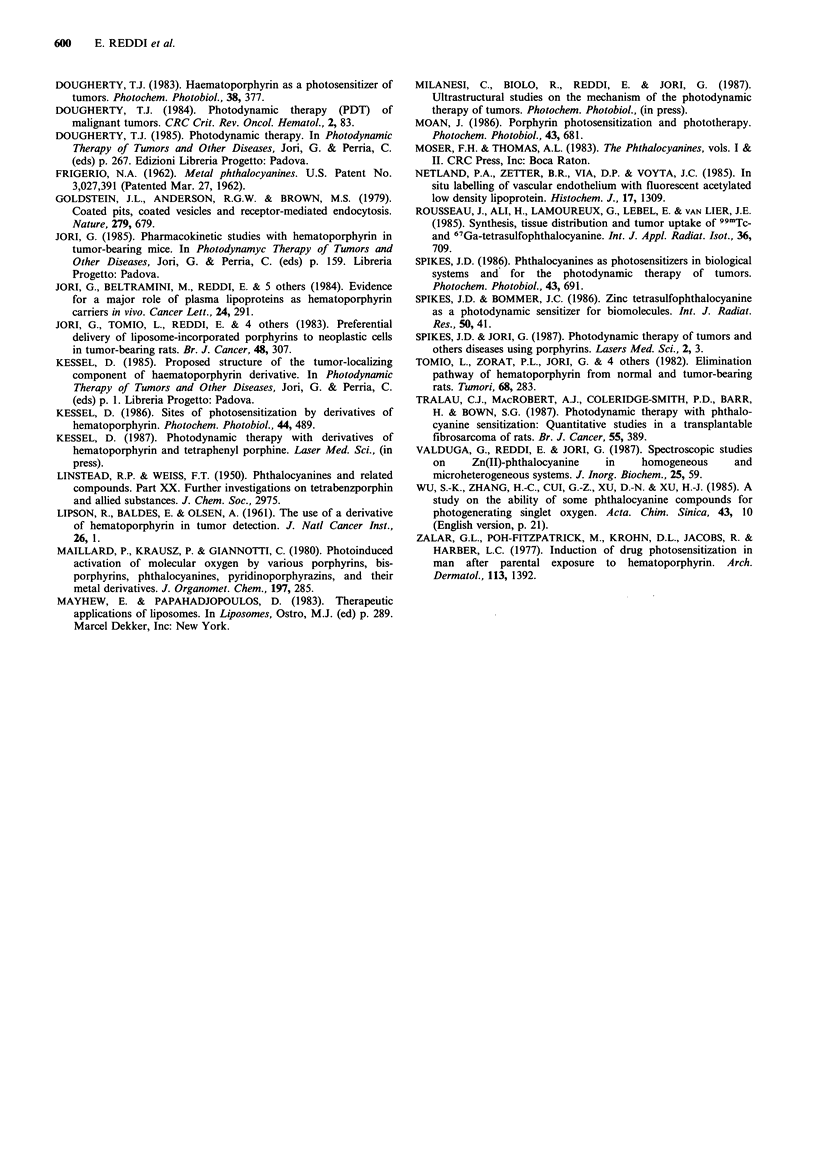# Pharmacokinetic studies with zinc(II)-phthalocyanine in tumour-bearing mice.

**DOI:** 10.1038/bjc.1987.247

**Published:** 1987-11

**Authors:** E. Reddi, G. Lo Castro, R. Biolo, G. Jori

**Affiliations:** Department of Biology, University of Padova, Italy.

## Abstract

Zn(II)-phthalocyanine (Zn-Pc) incorporated into unilamellar liposomes of dipalmitoylphosphatidylcholine has been injected intraperitoneally (0.5 mg kg-1) to BALB/c mice bearing a transplanted MS-2 fibrosarcoma. The drug is specifically transported by serum lipoproteins and cleared from the serum via the bile-gut pathway in a biphasic process: approximately 60% of Zn-Pc is eliminated with a serum half-life of approximately 9 hours, while the remaining aliquot is eliminated at a very slow rate. Several normal tissues take up the drug within 3 hours after administration but release it almost completely after 24-48 hours. On the other hand, the tumour shows a maximum concentration of Zn-Pc (approximately 0.6 microgram g-1 of tissue) after 18-24 hours; at this time, the ratio between the Zn-Pc levels in the tumour and the muscle (which represents the surrounding normal tissue) is approximately 7.5. The results are discussed in terms of a possible use of Zn-Pc as a photosensitizer in the photodynamic therapy of tumours.


 © The Macmillan Press Ltd., 1987

### Pharmacokinetic studies with zinc(II)-phthalocyanine in tumour-bearing mice

E. Reddi, G. Lo Castro, R. Biolo & G. Jori

*Department of Biology via Loredan 10, University of Padova, I-35131 Padova, Italy.*

**Summary** Zn(II)-phthalocyanine (Zn-Pc) incorporated into unilamellar liposomes of dipalmitoyl-phosphatidylcholine has been injected intraperitoneally (0.5 mg kg$^{-1}$) to BALB/c mice bearing a transplanted MS-2 fibrosarcoma. The drug is specifically transported by serum lipoproteins and cleared from the serum via the bile-gut pathway in a biphasic process: ~60% of Zn-Pc is eliminated with a serum half-life of ~9 hours, while the remaining aliquot is eliminated at a very slow rate. Several normal tissues take up the drug within 3 hours after administration but release it almost completely after 24–48 hours. On the other hand, the tumour shows a maximum concentration of Zn-Pc (~0.6 $\mu$g g$^{-1}$ of tissue) after 18–24 hours; at this time, the ratio between the Zn-Pc levels in the tumour and the muscle (which represents the surrounding normal tissue) is ~7.5. The results are discussed in terms of a possible use of Zn-Pc as a photosensitizer in the photodynamic therapy of tumours.

Haematoporphyrin and haematoporphyrin derivative (HpD) are presently used as sensitizers for the specific destruction of tumours by photodynamic therapy (PDT) (Moan, 1986; Spikes & Jori, 1987). Although the technique is being applied for the treatment of a variety of neoplasias at both the experimental and clinical level (Dougherty, 1984), PDT is facing some problems and/or limitations. Thus, HpD as it is prepared from Hp (Lipson *et al.*, 1961) is a complex mixture of porphyrins, whose composition is often not reproduced in different preparations and may change as a function of various experimental parameters (Bonnett *et al.*, 1981; Dougherty, 1983). So far, the most active components of HpD, which have been reported to possess an ether (Dougherty, 1985) or ester (Kessel, 1985) structure, have not been isolated in a pure form. Moreover, PDT is usually performed by irradiation with 620–630 nm light; unfortunately, the molar absorptivity of Hp or HpD in this wavelength range is very low ($<10^3$ M$^{-1}$ cm$^{-1}$).

Phthalocyanines, which are structurally similar to porphyrins, can potentially overcome some limitations of PDT; in particular, they exhibit a strong absorption ($\varepsilon \simeq 10^5$ M$^{-1}$ cm$^{-1}$) in the 680–700 nm region i.e., in correspondence of light wavelengths endowed with a high penetration power into biological tissues. The ability of phthalocyanines and some of their metal derivatives to act as efficient photosensitizers of simple biological substrates (Spikes & Bommer, 1986) and cultured cells (Ben-Hur & Rosenthal, 1985; Chan *et al.*, 1986) has been documented. Finally, Rousseau *et al.* (1985) have reported that tetra-sulphophthalocyanines (TSPc) can accumulate in a mammary adenocarcinoma in rats.

While tetrasulphophthalocyanines are suitable for *in vivo* studies because of their high water-solubility, serious problems may arise owing to the low degree of purity. Actually, the presently adopted procedures for sulfonation of phthalocyanines lead to mixtures of mono-, di-, tri- and tetra-sulphonated compounds (Linstead & Weiss, 1950; Moser & Thomas, 1983).

In general, underivatized phthalocyanines can be obtained with a very high purity and display a good efficiency in the generation of activated oxygen species (Maillard *et al.*, 1980; Wu *et al.*, 1985). Such water-insoluble dyes can be transported in the bloodstream via unilamellar liposomes (Valduga *et al.*, 1987); the latter carriers were shown to induce an efficient targeting of experimental tumours by water-insoluble porphyrins (Jori *et al.*, 1983), possibly through the preferential delivery of the incorporated drug to serum lipoproteins (Jori, 1985). In this paper, we describe the pharmacokinetic behaviour of liposome-bound Zn-phthalocyanine injected into BALB/c mice bearing a transplanted fibrosarcoma.

### Materials and methods

### *Chemicals*

$Zn^{2+}$-phthalocyanine (Zn-Pc) was supplied by Ciba-Geigy (Switzerland) and used without further purification. Sublimation of the sample under high vacuum showed a degree of purity of 97% (Valduga *et al.*, 1987). Dipalmitoyl-phosphatidylcholine (DPPC), over 98% pure, was a product of Sigma Chemical Co.; sodium dodecylsulphate (SDS) and Sephacryl S-300 were purchased from Merck and Pharmacia, respectively.

### *Animals and tumour*

Female mice of the BALB/c strain (20–25 g body wt) were obtained from Charles River (Como, Italy). The MS-2 fibrosarcoma was kindly supplied by Instituto Nazionale dei Tumori, Milan. It was implanted in the right hind leg of the mice by injection of $10^6$ cells suspended in 0.1 ml of PBS. The pharmacokinetic studies were begun at 8 days after tumour implantation, when its diameter was in the 0.7–1.0 cm range.

### *Pharmacokinetic studies*

The Zn-Pc was injected i.p. into tumour-bearing mice after incorporation of the dye into small unilamellar liposomes of DPPC following the procedure described by Valduga *et al.* (1987): The Zn-Pc/DPPC ratio was 1:36 on a molar basis, as assessed from the weighed amount of phospholipid and absorbance measurements at 680 nm for Zn-Pc ($\varepsilon = 1.68 \times 10^5$ M$^{-1}$ cm$^{-1}$ at 673 nm). The injected dose of Zn-Pc was 0.5 mg kg$^{-1}$ mouse body wt. At fixed times after Zn-Pc administration the mice were sacrificed and several tissues (tumour, muscle of the left hind leg, skin, liver, spleen, kidneys, lungs) were removed, washed with PBS and frozen until the analysis for Zn-Pc content was performed. Blood samples were collected from the same mice, centrifuged at 3,000 rpm for 15 min and the serum was analyzed for Zn-Pc content. The elimination pathway of Zn-Pc was studied by analysis of the faeces and urine collected from healthy Wistar albino rats, which had been injected with liposome-bound Zn-Pc at a dose of 0.5 mg kg$^{-1}$. Through-

Correspondence: E. Reddi.
Received 13 April 1987; and in revised form, 16 June 1987.

out the experiments the rats were maintained in metabolic cages with free access to standard dietary chow and the faeces and urines were collected until 48 h after the administration of Zn-Pc.

*Recovery of Zn-Pc from tissue and serum specimens*

Tissue samples (~200 mg of wet wt) were homogenized in 4 ml of 2% aqueous SDS as previously described (Jori *et al.*, 1983). The homogenate was magnetically stirred for 1 h at room temperature. The suspension thus obtained was centrifuged at 3,000 rpm for 10 min; the supernatant was collected and its Zn-Pc content ($\mu$g drug ml$^{-1}$ solution) was estimated by fluorescence measurements with a Perkin-Elmer MPF4 apparatus. The results of Zn-Pc recovery were finally referred to an identical weight of each tissue, i.e., 1 g. The sample was placed in quartz cuvettes of 1 cm optical path and its 600 nm-excited fluorescence was recorded in the 630–740 nm spectral interval at a right angle to the incident light beam; in this way, the contribution of scattered light could be more easily subtracted. To minimize inner filter effects, the absorbance of the analyzed solutions at the excitation wavelength was kept below 0.1. The fluorescence intensity data were converted into Zn-Pc concentration by interpolation with a calibration plot built with known amounts of Zn-Pc in 2% SDS; preliminary studies (Valduga *et al.*, 1987) have shown that under our experimental conditions Zn-Pc is embedded in a monomeric state within the surfactant micelles. In preliminary studies, the pellet remaining after removal of the supernatant was resuspended in 2% SDS (4 ml) and processed as above described; only negligible amounts of Zn-Pc fluorescence were observed.

Serum samples were diluted with suitable volumes of 2% aqueous SDS, so that the absorbance at 600 nm was lower than 0.1. The fluorescence emission of Zn-Pc was then determined as described above. The Zn-Pc fluorescence obtained from tissue extracts was corrected for the contribution of a 600 nm-excitable background fluorescence as observed in tissue extracts from control mice.

*Chromatographic studies*

Serum samples were chromatographed on a column (1.7 x 140 cm) of Sephacryl S-300, which had been equilibrated with 0.01 M phosphate buffer at pH 7.4, containing 0.15 M NaCl. The column was eluted at a flow-rate of 26 ml h$^{-1}$ and 2.5 ml-fractions were collected. The fraction collector was connected to a 2238 LKB UV-cord and the protein content was continuously recorded by monitoring the absorbance of the eluate at 280 nm. The collected fractions were also analyzed for their Zn-Pc content by measuring the intensity at 680 nm of the 600 nm-excited fluorescence. This value was found to be proportional to the integrated area of the whole fluorescence spectrum.

### Results

The serum concentration of i.p.-injected Zn-Pc reaches a maximum value at ~3 h after administration (Figure 1). About 60% of Zn-Pc is eliminated with a half-life of ~9 h, whereas the serum levels of the remaining drug undergo an approximately exponential decrease at a low rate. Closely similar behaviour has been reported for HpD administered to patients (Zalar *et al.*, 1977). The rapid clearance of Zn-Pc from the serum appears to occur almost exclusively via the bile-gut pathway. Actually, analyses of faeces eliminated from Wistar rats which had been maintained in metabolic cages for 48 h after i.p. administration of the drug showed the presence of a total amount of Zn-Pc ranging between 109 and 127 $\mu$g as compared to a total recovery ranging between 0.66 and 0.81 $\mu$g of Zn-Pc from the urine collected over the same period of time. Similarly, Tomio *et al.* (1982) observed

**Figure 1** Time-dependence of Zn-Pc concentration in the serum of BALB/c mice bearing the MS-2 fibrosarcoma. The mice have been injected with 0.5 mg kg$^{-1}$ of Zn-Pc incorporated into DPPC liposomes.

**Figure 2** Chromatogram of the serum of BALB/c mice 24 h after the administration of liposomal Zn-Pc at a dose of 0.5 mg kg$^{-1}$. The chromatogram show the absorbance at 280 nm (○—○) and the fluorescence emission at 680 nm (●—●).

that at least 98% of the totally eliminated haematoporphyrin from Wistar rats is recovered in the faeces.

Column chromatography of mouse serum on Sephacryl S-300 allows us to separate three protein fractions (Figure 2). Typically, at 24 h after i.p.-administration to tumour-bearing mice, the Zn-Pc fluorescence is specifically associated with the second protein peak. This peak appears to be mainly constituted by the lipoprotein class as determined by the estimation of its cholesterol and phospholipid content (such analyses were carried out as described by Jori *et al.*, 1984). The specific association of Zn-Pc with this group of serum proteins is independent of the time after administration: chromatographic patterns qualitatively identical with those shown in Figure 2 were obtained for sera taken at various times between 1 h and 1 week after administration of Zn-Pc.

The maximum concentration of Zn-Pc in the serum corresponds with the maximum uptake of the drug by several normal tissues, including liver, kidneys, spleen, lungs and muscle (Figures 3 and 4). In Figure 4 we show comparatively the time-dependency of Zn-Pc recovery from the MS-2 fibrosarcoma and the muscle, i.e., the surrounding normal tissue. All the recovery data represent the average of the values obtained by separate analyses of the tissues taken from groups of at least three animals for each time, the largest deviation from the reported values being 20%.

### Discussion

Some water-soluble metal complexes of TSPc have been shown to be accumulated in significant amounts by brain tumours implanted into mice (Frigerio, 1962) or other kinds

**Figure 3** Uptake and release of Zn-Pc in lungs (●—●), kidneys (■—■), liver (○—○) and spleen (△—△) of BALB/c mice after injection of 0.5 mg kg$^{-1}$ Zn-Pc.

**Figure 4** Uptake and release of Zn-Pc in tumour (●—●) and muscle (○—○) of mice after injection of 0.5 mg kg$^{-1}$ Zn-Pc.

of tumours in rats (see, for a recent review, Spikes, 1986). The problem of the selectivity of TSPc uptake by tumour tissues as compared with the tissue where the tumour growth has been addressed by Tralau *et al.* (1987): their results, although preliminary, would indicate a similar tumour-specificity of TSPc in comparison with HpD.

The data presented in this paper show that the water-insoluble Zn-Pc is also a good tumour-localizer, at least in our animal model. In particular, maximal tumour concentrations of Zn-Pc are found at ~18–24 h after i.p. administration of the drug, while after this time period most Zn-Pc has been released from normal tissues. Thus, at 24 h, the ratio of Zn-Pc content between the tumour and the muscle, i.e., the surrounding normal tissue, is ~7.5. One should remember, however, that the muscle does not represent the normal tissue where the MS-2 fibrosarcoma originates or grows.

Several indications suggest that TSPc and Zn-Pc are transported and delivered to tumours by at least partially different mechanisms. Thus i.v. injected TSPc are rapidly (1–3 h) accumulated by a fibrosarcoma implanted s.c. in the flank and completely disappear from the serum within 3 h (Tralau *et al.*, 1987). It is likely that the latter dyes are mainly bound by serum albumin, as it has been observed for tetrasulphonated porphyrins (Kessel, 1987; Milanesi *et al.*, 1987); albumin does not enter tumour cells, hence the associated drug is present in the vascular stroma of the neoplastic tissue at least for relatively short (24 h) periods of time after its administration. On the contrary, Zn-Pc is specifically transported by lipoproteins: this fact is not surprising in view of the highly lipophilic nature of the drug, as well as the tendency of the liposome carriers of Zn-Pc to fuse with the lipid matrix of lipoproteins and to release the entrapped drug to lipoproteins (Mayhew & Papahadjopoulos, 1983). Now, lipoproteins, especially LDL, preferentially interact with neoplastic and endothelial cells of tumour tissues through a receptor-mediated endocytosis, so that the drug is delivered from inside the cells (Goldstein *et al.*, 1979; Netland *et al.*, 1985). The high efficiency of Zn-Pc binding by lipoproteins can thus be correlated with the high amounts of drug accumulated by the tumour tissue (~0.6 μg g$^{-1}$ tissue) in spite of the low injected doses, i.e., 0.5 mg kg$^{-1}$ body wt. The above considerations may explain the different pharmacokinetic behaviour between Zn-Pc and TSPc, although both types of phthalocyanines can act as good tumour-localizing agents. Certainly, the control of such a behaviour has a critical importance for achieving a satisfactory degree of selectivity in the targeting of tumour tissues by photosensitizing agents (Jori, 1985).

In this connection, Zn-Pc has the distinct advantage over HpD and photofrin II of a more homogeneous distribution among serum proteins. It has been shown that both haematoporphyrin (Jori *et al.*, 1984) and HpD (Kessel, 1987) are transported by at least three different classes of serum proteins; this fact may explain the heterogeneous and time-dependent distribution of these porphyrins in tumour tissues (Kessel, 1986).

On the other hand, detectable amounts of HpD and Zn-Pc persist in the serum for some weeks after their administration. This circumstance has been claimed (Zalar *et al.*, 1977) to be correlated with the prolonged skin photosensitivity, which represents one major side effect of the photodynamic therapy of tumours.

In order to obtain some information on this point, we estimated the skin concentration of Zn-Pc in mice at selected times. Although there was some degree of individual variability of the recovery data, in no case was the skin concentration of Zn-Pc between 1 h and 168 h after administration found to be >0.1 μg g$^{-1}$ tissue. Therefore, one would not expect a significant level of skin photosensitization by Zn-Pc. The relatively inefficient accumulation of Zn-Pc by mouse skin may again be a consequence of the mechanisms involved in the transport of this drug in the bloodstream. We have observed that, upon *in vitro* incubation of human serum with Zn-Pc incorporated into DPPC liposomes, a selective association of the phthalocyanine with lipoproteins takes place: at least 70% of the drug is bound by HDL (Reddi *et al.*, unpublished results). HDL are known to be responsible for the prolonged serum persistence of a major fraction of i.v. injected haematoporphyrin (Barel *et al.*, 1986).

In summary, the present findings encourage us to test the efficiency of liposome-carried Zn-Pc as a phototherapeutic agent: although the actual clinical use of this drug requires thorough toxicological studies. Experiments aimed at assessing the optimal parameters for photodynamic therapy of tumours with Zn-Pc are in progress in our laboratory.

This work received financial support in part from Consiglio Nazionale delle Ricerche (Italy), under the special project 'Oncologia', contract No. 86.00451.44, and in part from Teclas (Laser Technologies, Lugano, Switzerland). One of us (RB) is recipient of a fellowship given by Teclas.

### References

BAREL, A., JORI, G., PERIN, A., ROMANDINI, P., PAGNAN, A. & BIFFANTI, S. (1986). Role of high-, low- and very low-density lipoproteins in the transport and tumor-delivery of hematoporphyrin *in vivo*. *Cancer Lett.*, **32**, 145.

BEN-HUR, E. & ROSENTHAL, I. (1985). The phthalocyanines: A new class of mammalian cell photosensitizers with a potential for cancer phototherapy. *Int. J. Radiat. Biol.*, **47**, 145.

BONNETT, R., RIDGE, R.J. & SCOURIDES, P.A. (1981). On the nature of hematoporphyrin derivative. *J.C.S. Perkin I*, 3135.

CHAN, W.S., SVENSEN, R., PHILLIPS, D. & HART, I.R. (1986). Cell uptake, distribution and response to aluminium chloro sulphonated phthalocyanine, a potential anti-tumor photosensitizer. *Br. J. Cancer*, **53**, 255.

DOUGHERTY, T.J. (1983). Haematoporphyrin as a photosensitizer of tumors. *Photochem. Photobiol.*, **38**, 377.

DOUGHERTY, T.J. (1984). Photodynamic therapy (PDT) of malignant tumors. *CRC Crit. Rev. Oncol. Hematol.*, **2**, 83.

DOUGHERTY, T.J. (1985). Photodynamic therapy. In *Photodynamic Therapy of Tumors and Other Diseases*, Jori, G. & Perria, C. (eds) p. 267. Edizioni Libreria Progetto: Padova.

FRIGERIO, N.A. (1962). *Metal phthalocyanines.* U.S. Patent No. 3,027,391 (Patented Mar. 27, 1962).

GOLDSTEIN, J.L., ANDERSON, R.G.W. & BROWN, M.S. (1979). Coated pits, coated vesicles and receptor-mediated endocytosis. *Nature*, **279**, 679.

JORI, G. (1985). Pharmacokinetic studies with hematoporphyrin in tumor-bearing mice. In *Photodynamyc Therapy of Tumors and Other Diseases*, Jori, G. & Perria, C. (eds) p. 159. Libreria Progetto: Padova.

JORI, G., BELTRAMINI, M., REDDI, E. & 5 others (1984). Evidence for a major role of plasma lipoproteins as hematoporphyrin carriers *in vivo*. *Cancer Lett.*, **24**, 291.

JORI, G., TOMIO, L., REDDI, E. & 4 others (1983). Preferential delivery of liposome-incorporated porphyrins to neoplastic cells in tumor-bearing rats. *Br. J. Cancer*, **48**, 307.

KESSEL, D. (1985). Proposed structure of the tumor-localizing component of haematoporphyrin derivative. In *Photodynamic Therapy of Tumors and Other Diseases*, Jori, G. & Perria, C. (eds) p. 1. Libreria Progetto: Padova.

KESSEL, D. (1986). Sites of photosensitization by derivatives of hematoporphyrin. *Photochem. Photobiol.*, **44**, 489.

KESSEL, D. (1987). Photodynamic therapy with derivatives of hematoporphyrin and tetraphenyl porphine. *Laser Med. Sci.*, (in press).

LINSTEAD, R.P. & WEISS, F.T. (1950). Phthalocyanines and related compounds. Part XX. Further investigations on tetrabenzporphin and allied substances. *J. Chem. Soc.*, 2975.

LIPSON, R., BALDES, E. & OLSEN, A. (1961). The use of a derivative of hematoporphyrin in tumor detection. *J. Natl Cancer Inst.*, **26**, 1.

MAILLARD, P., KRAUSZ, P. & GIANNOTTI, C. (1980). Photoinduced activation of molecular oxygen by various porphyrins, bis-porphyrins, phthalocyanines, pyridinoporphyrazins, and their metal derivatives. *J. Organomet. Chem.*, **197**, 285.

MAYHEW, E. & PAPAHADJOPOULOS, D. (1983). Therapeutic applications of liposomes. In *Liposomes*, Ostro, M.J. (ed) p. 289. Marcel Dekker, Inc: New York.

MILANESI, C., BIOLO, R., REDDI, E. & JORI, G. (1987). Ultrastructural studies on the mechanism of the photodynamic therapy of tumors. *Photochem. Photobiol.*, (in press).

MOAN, J. (1986). Porphyrin photosensitization and phototherapy. *Photochem. Photobiol.*, **43**, 681.

MOSER, F.H. & THOMAS, A.L. (1983). *The Phthalocyanines*, vols. I & II. CRC Press, Inc: Boca Raton.

NETLAND, P.A., ZETTER, B.R., VIA, D.P. & VOYTA, J.C. (1985). In situ labelling of vascular endothelium with fluorescent acetylated low density lipoprotein. *Histochem. J.*, **17**, 1309.

ROUSSEAU, J., ALI, H., LAMOUREUX, G., LEBEL, E. & VAN LIER, J.E. (1985). Synthesis, tissue distribution and tumor uptake of $^{99m}$Tc- and $^{67}$Ga-tetrasulfophthalocyanine. *Int. J. Appl. Radiat. Isot.*, **36**, 709.

SPIKES, J.D. (1986). Phthalocyanines as photosensitizers in biological systems and for the photodynamic therapy of tumors. *Photochem. Photobiol.*, **43**, 691.

SPIKES, J.D. & BOMMER, J.C. (1986). Zinc tetrasulfophthalocyanine as a photodynamic sensitizer for biomolecules. *Int. J. Radiat. Res.*, **50**, 41.

SPIKES, J.D. & JORI, G. (1987). Photodynamic therapy of tumors and others diseases using porphyrins. *Lasers Med. Sci.*, **2**, 3.

TOMIO, L., ZORAT, P.L., JORI, G. & 4 others (1982). Elimination pathway of hematoporphyrin from normal and tumor-bearing rats. *Tumori*, **68**, 283.

TRALAU, C.J., MACROBERT, A.J., COLERIDGE-SMITH, P.D., BARR, H. & BOWN, S.G. (1987). Photodynamic therapy with phthalocyanine sensitization: Quantitative studies in a transplantable fibrosarcoma of rats. *Br. J. Cancer*, **55**, 389.

VALDUGA, G., REDDI, E. & JORI, G. (1987). Spectroscopic studies on Zn(II)-phthalocyanine in homogeneous and microheterogeneous systems. *J. Inorg. Biochem.*, **25**, 59.

WU, S.-K., ZHANG, H.-C., CUI, G.-Z., XU, D.-N. & XU, H.-J. (1985). A study on the ability of some phthalocyanine compounds for photogenerating singlet oxygen. *Acta. Chim. Sinica*, **43**, 10 (English version, p. 21).

ZALAR, G.L., POH-FITZPATRICK, M., KROHN, D.L., JACOBS, R. & HARBER, L.C. (1977). Induction of drug photosensitization in man after parental exposure to hematoporphyrin. *Arch. Dermatol.*, **113**, 1392.